# Targeting cathepsin C ameliorates murine acetaminophen-induced liver injury

**DOI:** 10.7150/thno.96092

**Published:** 2024-05-13

**Authors:** Jessica Raith, Malte Bachmann, Sina Gonther, Hendrik Stülb, Ali A. Aghdassi, Christine T. N. Pham, Heiko Mühl

**Affiliations:** 1 pharmazentrum frankfurt/ZAFES, Institute of General Pharmacology and Toxicology, Faculty of Medicine, Goethe University Frankfurt, Frankfurt am Main, Germany.; 2 Department of Medicine A, University Medicine Greifswald, Greifswald, Germany.; 3 John Cochran VA Medical Center, Saint Louis, MO, USA; Department of Medicine, Division of Rheumatology and the Department of Surgery, Section of Vascular Surgery, Washington University School of Medicine, Saint Louis, MO, USA.

**Keywords:** Acetaminophen, Acute Liver Injury, Inflammation, Cathepsin C, AZD7986

## Abstract

Acetaminophen (APAP) overdosing is a major cause of acute liver failure worldwide and an established model for drug-induced acute liver injury (ALI). While studying gene expression during murine APAP-induced ALI by 3'mRNA sequencing (massive analysis of cDNA ends, MACE), we observed splenic mRNA accumulation encoding for the neutrophil serine proteases cathepsin G, neutrophil elastase, and proteinase-3 - all are hierarchically activated by cathepsin C (CtsC). This, along with increased serum levels of these proteases in diseased mice, concurs with the established phenomenon of myeloid cell mobilization during APAP intoxication.

**Objective:** In order to functionally characterize CtsC in murine APAP-induced ALI, effects of its genetic or pharmacological inhibition were investigated.

**Methods and Results:** We report on substantially reduced APAP toxicity in CtsC deficient mice. Alleviation of disease was likewise observed by treating mice with the CtsC inhibitor AZD7986, both in short-term prophylactic and therapeutic protocols. This latter observation indicates a mode of action beyond inhibition of granule-associated serine proteases. Protection in CtsC knockout or AZD7986-treated wildtype mice was unrelated to APAP metabolization but, as revealed by MACE, realtime PCR, or ELISA, associated with impaired expression of inflammatory genes with proven pathogenic roles in ALI. Genes consistently downregulated in protocols tested herein included *cxcl2*, *mmp9*, and *angpt2*. Moreover, *ptpn22*, a positive regulator of the toll-like receptor/interferon-axis, was reduced by targeting CtsC.

**Conclusions:** This work suggests CtsC as promising therapeutic target for the treatment of ALI, among others paradigmatic APAP-induced ALI. Being also currently evaluated in phase III clinical trials for bronchiectasis, successful application of AZD7986 in experimental APAP intoxication emphasizes the translational potential of this latter therapeutic approach.

## Introduction

Acetaminophen (also paracetamol or N-acetyl-para-aminophenol, APAP) is an over-the-counter available analgesic that is widely used to treat mild-to-moderate pain and fever but also characterized by a narrow therapeutic index - mirrored by its classification as a major cause of acute liver failure worldwide 1, 2. Initial hepatocyte necrosis after APAP overdosing is directly induced by N-acetyl-para-benzoquinone imine (NAPQI), a cytochrome P450 (Cyp) 1A2/Cyp2E1-dependent APAP metabolite, acting through mitochondrial protein adduct formation 3-7. Whereas NAPQI is evidently at the root of APAP intoxication, the functional role of simultaneously induced hepatic necroinflammation is multilayered 7. On the one hand, specific inflammatory pathways engaged by cytokines such as interleukin (IL)-17 8, 9, IL-18 10, 11, and interferon (IFN)-γ 12, 13 are consistently found to contribute to APAP-mediated liver pathology. On the other hand, current data likewise suggest that inflammation can support the resolution of APAP-induced acute liver injury (ALI) 7, 14, for example by activating pro-regenerative hepatocyte signaling through IL-6 15 and C5a/C5aR1 16. This dichotomy also extends to the function of neutrophils which have been reported to promote hepatic injury during APAP intoxication 17, 18, for example by serving as a source of pathogenic IFNγ 19. Contrariwise, neutrophils are also supposed to promote resolution of APAP-induced ALI by removing cell debris 20 and by supporting the generation of pro-resolving macrophages 21. Most recent data suggest that effects of neutrophils during APAP intoxication are determined by the degree of the given hepatic damage 22, a concept that would unify aforementioned Janus-faced properties of neutrophils as detected in different studies.

Cathepsin C (CtsC, also dipeptidyl peptidase-1) is a highly conserved, ubiquitously expressed lysosomal cysteine dipeptidyl aminopeptidase that mediates constitutive activation of granule-associated serine proteases in hematopoietic precursor cells. Those proteases include the so-called neutrophil serine proteases (NSPs), specifically cathepsin G (CtsG), neutrophil elastase (NE; murine gene name, *elane*), and proteinase-3 (Prtn3), as well as chymases, tryptases, and granzymes. They are supposed to mediate key functions in diverse immune cells such as neutrophils, monocytes, mast cells, basophils, natural killer cells, and cytotoxic T cells 23-25. In terms of organ distribution, CtsC is, besides in bone marrow, detectable with high constitutive expression levels e.g. in spleen, lung, and liver tissues 25, 26. Specifically, CtsC enzyme activity was recognized early on in liver homogenates of healthy rodents and ascribed to hepatocytes and Kupffer cells 27. Just recently this observation has been supported by immunohistochemical CtsC analysis performed on livers of C57BL/6 mice and by the observation that CtsC protein is constitutively expressed in human HepG2 hepatoma cells 28. Because CtsC is generally known to be a proximal regulator of proteases involved in acute inflammation, we set out to investigate CtsC as a potential pharmacological target in APAP intoxication.

## Materials and Methods

### Animals and experimental APAP-induced ALI

**Figure 1:**
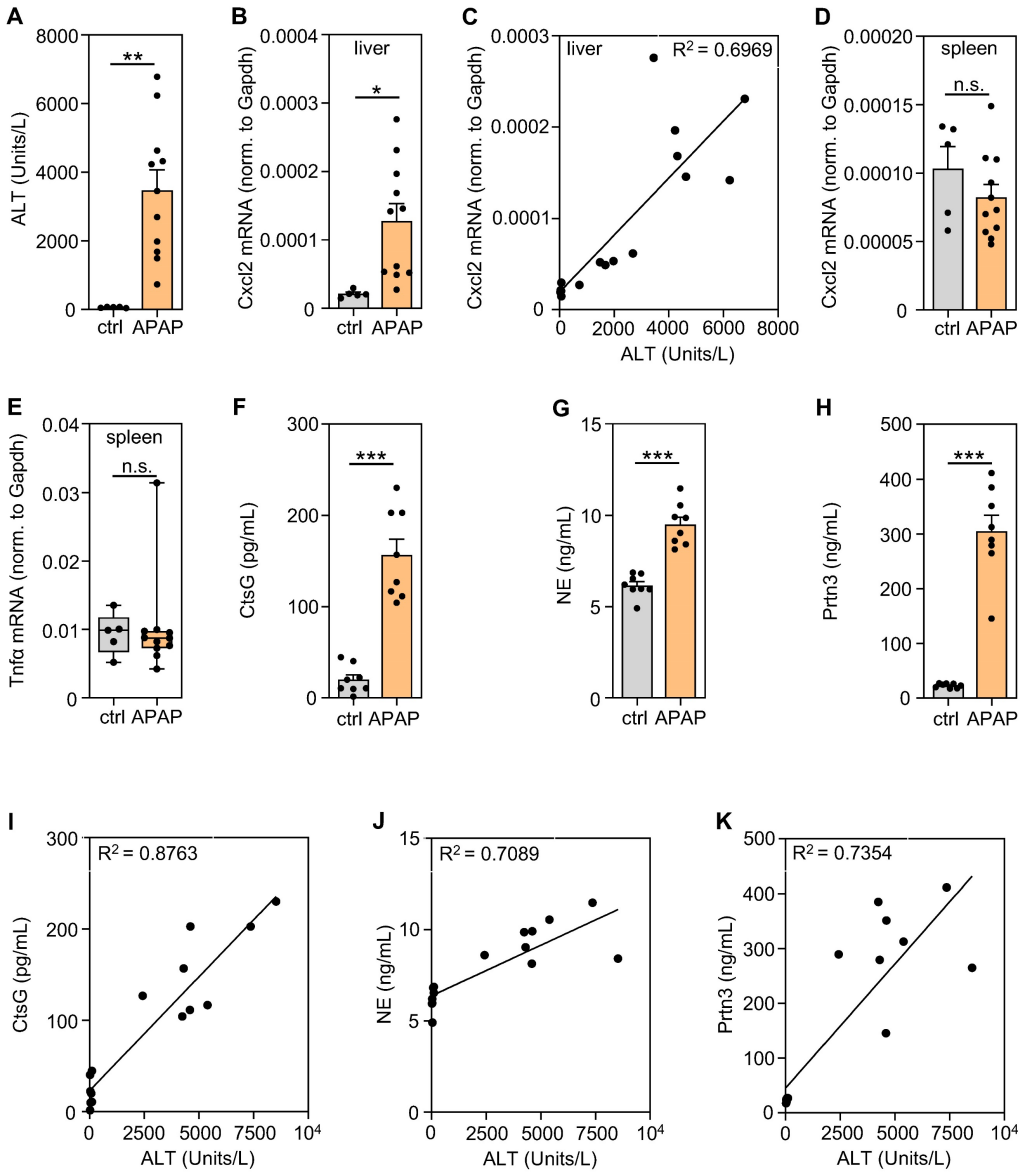
** Significantly increased levels of serum NSPs in APAP-induced ALI and their correlation with disease severity. A-E** Male C57BL/6J mice received either 0.9% NaCl (ctrl, n = 5) or APAP at 300 mg/kg (n = 11). After 30 h, splenic/hepatic tissues and sera were analyzed. **A** To assess liver damage, serum ALT levels were determined (**p < 0.01). **B** Hepatic mRNA expression of Cxcl2 in these same mice was analyzed using realtime PCR and results showed a strong correlation to the linked ALT levels (**C**). Moreover, splenic mRNA expression of Cxcl2 (**D**) and Tnfα (**E**) was analyzed using realtime PCR. **B, D-E** Target mRNA normalized to Gapdh is shown as absolute values (*p < 0.05). **F-H** Male C57BL/6J mice received either 0.9% NaCl or APAP at 300 mg/kg (each group n = 8). After 30 h, serum levels of CtsG (**F**), NE (**G**), and Prtn3 (**H**) were determined by ELISA (***p < 0.001). **I-K** In these same serum samples, CtsG (**I**), NE (**J**), and Prtn3 (**K**) strongly correlated with their linked serum ALT levels. Statistical analysis: **A-B, D, F-H**, raw data were analyzed by unpaired Student´s *t*-test and are shown as means ± SEM; **E**, raw data were analyzed by Mann-Whitney-U-test and are shown as boxplots; **C, I-K,** R^2^ was calculated by simple linear regression; n.s., not statistically significant.

Animal experiments were conducted at the Zentrale Forschungseinrichtung (ZFE, Faculty of Medicine, Goethe-University Frankfurt am Main, Frankfurt am Main, Germany) using male C57BL/6J mice (9-11 weeks old) in accord with the recommendations of the Animal Protection Agency of the Federal State of Hessen (Regierungspräsidium Darmstadt, Germany). The protocols applied herein were approved by the Regierungspräsidium Darmstadt (FU1230, FU2021, FU2036). All mice were housed in type II-long-IVC under a 12 h light-dark cycle with access to food and water *ad libitum*. Mice underwent a 10 h overnight fasting period (with free access to water) prior to APAP (or 0.9% NaCl as vehicle) administration; details are outlined below. For splenic 3'mRNA sequencing experiments and related Figures 1A-E, Figure S1AB, and Figure S2A, wildtype (WT) male C57BL/6J mice (maintained and breed at MfD Diagnostics (Wendelsheim, Germany)) were initially obtained from Prof. Jörg Köhl (Institute for Systemic Inflammation Research, University of Lübeck, Lübeck, Germany). CtsC^-/-^ (KO) mice with a C57BL/6J background were generated by Prof. Christine Pham 29, 30: these mice were originally on a 129/SVJ background displaying a defective tyrosinase gene and an albino phenotype. CtsC-KO mice were fully backcrossed to C57BL/6J (99% congenic) which was verified by microsatellite polymerase chain reaction (PCR). Because *ctsc* and *tyrosinase* are closely linked on chromosome 7 the albino phenotype prevailed in CtsC-KO mice. We generated suitable WT mice by crossing CtsC-KO mice with WT C57BL/6J mice (Charles River Laboratories, Sulzfeld, Germany). Heterozygotes were further crossed to obtain homozygote CtsC-KO and corresponding WT mice - those mice strains were further breed in the ZFE. Genetic identities of all CtsC-KO and corresponding WT mice were confirmed by PCR. Of note, APAP is a substrate of tyrosinase producing the minor APAP metabolite 3'-hydroxyacetaminophen and its downstream reaction product 4-acetamido-o-benzoquinone 31. However, this pathway does not contribute to APAP-induced ALI because 3'-hydroxyacetaminophen is not hepatotoxic in mice 32, 33. All C57BL/6J mice used to study AZD7986 and those used in Figure S2B-D were purchased from Charles River Laboratories.

For experimentation, male mice were fasted overnight for 10 h with free access to water. Instantly thereafter, APAP intoxication was initiated as previously described 34. Briefly, mice obtained i.p. injections of either body tempered 0.9% NaCl solution (B. Braun, Melsungen, Germany) or 300 mg/kg APAP (Sigma-Aldrich, Taufkirchen, Germany) dissolved in warm 0.9% NaCl. Mice receiving 0.9% NaCl alone are referred to as controls (ctrl). Thereafter, all mice had access to food and water *ad libitum*. As outlined in the Figures and their legends, mice received repeatedly 5 mg/kg of the CtsC Inhibitor AZD7986 35 (MedChemExpress (Hölzel diagnostica), Cologne, Germany) dissolved using 0.5% hydroxypropyl methylcellulose and 0.1% Tween 80 (MedChemExpress) in 0.1 M citrate buffer (pH = 3; Sigma-Aldrich). This mixture (without AZD7986) likewise served as vehicle control administered to ctrl mice (also denoted as vehicle-AZD7986) in respective experiments. Experimental details are specified in the legends. Experiments were terminated after the indicated time periods. Upon the completion of the experimental protocol, mice underwent a brief isoflurane (Abbott, Wiesbaden, Germany) anesthesia before being sacrificed through cervical dislocation. Blood was obtained from the retroorbital venous plexus, and the resultant serum was stored at -80 °C. For tissue processing, livers underwent perfusion with PBS. Subsequently, specimens were incubated overnight in 4.5% buffered formalin to enable histological analysis on paraffin-embedded sections. To evaluate tissue mRNA and protein expression, specimens were snap-frozen and stored at -80 °C.

### Biochemical and histological determination of liver injury

Serum ALT activity was determined according to the manufacturer's instructions (Reflotron; Roche Diagnostics GmbH, Mannheim, Germany). Formalin-fixed tissue samples were embedded in paraffin, followed by the generation of liver sections (4 μm) that were further processed by H&E staining. Slides were scanned using NanoZoomer S360 (Hamamatsu Photonics Deutschland GmbH, Herrsching am Ammersee, Germany) and analyzed with NDPview2 (Hamamatsu Photonics Deutschland GmbH). Histopathological liver injury was quantified in blinded manner by Keyence BZ-II Analyzer software (Neu-Isenburg, Germany) with necrotic areas being expressed as [% of total liver section].

### Determination of mRNA expression levels by realtime PCR

Total RNA was obtained by Tri-Reagent (Sigma-Aldrich) and transcribed using random hexameric primers (Qiagen, Hilden, Germany) and Moloney virus reverse transcriptase (Thermo Fisher Scientific, Darmstadt, Germany). RNA isolates were routinely treated with RNase-free DNase I (Roche Diagnostics GmbH) prior to reverse transcription. During realtime PCR, changes in fluorescence were caused by the Taq polymerase degrading the probe containing a fluorescent dye (glyceraldehyde-3-phosphate dehydrogenase (Gapdh): VIC, all other probes: FAM). Commercially available reagents (Thermo Fisher Scientific) were used for the determination of murine mRNA expression: Gapdh (4352339E), angiopoietin-2 (Angpt2, Mm00545822_m1), CtsC (Mm00515580_m1), CtsG (Mm00456011_m1), Cxcl2 (Mm00436450_m1), matrix metalloproteinase-9 (Mmp9, Mm00442991_m1), NE (Elane; Mm00469310_m1), Prtn3 (Mm00478323_m1), tyrosine-protein phosphatase non-receptor type 22 (Ptpn22, Mm00501246_m1), and tumor necrosis factor-α (Tnfα, Mm00443258_m1). The assay-mix was purchased from Nippon Genetics (Düren, Germany). Realtime PCR was performed according to the manufacturers' instructions using QuantStudio 3 Sequence Detector (Thermo Fisher Scientific): one initial step at 95 °C (2 min) was followed by 40 cycles at [95 °C (5 s) and 62 °C (30 s)]. Detection of the dequenched probe, calculation of threshold cycles (Ct values) and data analysis was performed by the Sequence Detector software. Changes in mRNA expression compared to unstimulated control and normalized to Gapdh are shown as 2^-dCT^ (absolute values).

### 3′mRNA sequencing by massive analysis of cDNA ends (MACE)

After treatment of samples using the DNase I recombinant RNase-free Kit (Roche Diagnostics), RNA specimens underwent a quality check by electrophoresis. Of note, in order to detect low abundant transcripts in specimens dominated by blood cells, removal of globin RNA is recommended. This procedure can associate with some reduction of RNA quality 36, 37. However, in the context of the present study aiming to detect proinflammatory gene expression in the spleen that may feedback on liver pathology (Figure S2A), we decided to leave splenic RNA specimens as untouched as possible at the expense of some sensitivity. Pooled RNA samples were generated from selected experimental groups as outlined in the “Results” section and used for further analysis by 3′mRNA sequencing. MACE, a 3′mRNA sequencing approach using Illumina reads of fragments originated from 3′mRNA ends 38, was performed at GenXPro GmbH (Frankfurt, Germany). To that end, the MACE-kit v.2 (Figure S2A) or Rapid MACE-Seq kit (Figure 4A, Table S1) were run according to GenXPro. RNA was fragmented and enriched polyadenylated mRNA underwent poly-A specific reverse transcription and template-switch-based second strand syntheses followed by competitive PCR. Duplicate reads verified by implemented unique molecular identifiers (TrueQuant IDs) were eliminated from the raw dataset. Using cutadapt (https://github.com/marcelm/cutadapt/), low-quality sequence bases were eliminated. Poly(A)-tails were clipped by an in-house Python-Script. After mapping the reads on the mouse reference genome (mm10), transcripts were quantified by HTSeq. Identification of differentially expressed genes was achieved by DESeq2 39. Genes with “0 counts” were set to “1 count” in order to allow further calculation of the respective “fold-induction”. Splenic gene expression (Figure S2A): further analysis of MACE results using UniProt (https://www.uniprot.org) was performed as outlined in the “Results” section. Analysis of hepatic gene expression by MACE (Figure 4A, Table S1): GO term analysis of “biological processes” was performed using GOrilla 40. Evaluation was performed as outlined in the “Results” section.

3'mRNA sequencing (MACE) data (Figure S2A, Figure 4A, Table S1) were deposited in NCBI's Gene Expression Omnibus (GEO) under the following accession numbers: GSE263675 (Figure S2A) and GSE263673 (Figure 4A, Table S1).

### Analysis of reduced glutathione (GSH) in liver tissue

For the determination of hepatic GSH content, snap-frozen liver tissue was homogenized in 5-Sulfosalicylic acid buffer (Sigma-Aldrich). GSH content was determined using a colorimetric GSH assay kit (Sigma-Aldrich) according to the manufacturer's instructions. Hepatic GSH content is expressed as μmol/g of liver tissue.

### Analysis of hepatic protein expression by enzyme-linked immunosorbent assay (ELISA)

Serum concentrations of murine NE (Quantikine assay; R&D Systems, Wiesbaden, Germany), CtsG (Novus Biologicals, Wiesbaden, Germany), and Prtn3 (Aviva Systems Biology/Biozol, Eching, Germany) as well as protein levels of Cxcl2, Mmp9, Angpt2 (Quantikine assays; R&D Systems), and Ptpn22 (Abbexa/Hölzel diagnostica) in murine liver lysates were determined by ELISA according to the manufacturers' instructions. For detection of Angpt2 in liver tissue, specimens were homogenized in sample diluent concentrate-2 (R&D Systems); for Cxcl2, Mmp9 and Ptpn22, specimens were homogenized in lysis buffer (1 mM CaCl_2_, 150 mM NaCl, 25 mM Tris-HCl (pH 7.4), 1% Triton X-100). Sample diluent concentrate-2 and lysis buffer were additionally supplemented with protease inhibitor cocktail (Roche Diagnostics) and DTT, Na_3_VO_4_, PMSF (each 1 mM) and 20 mM NaF. ELISA standards and blanks were prepared in corresponding dilutions of supplemented sample diluent concentrate-2 or lysis buffer, respectively.

### Immunoblot analysis

Tissue homogenates were generated as previously described 34. Briefly, liver homogenates were generated using lysis buffer (1 mM CaCl_2_, 150 mM NaCl, 25 mM Tris-Cl (pH 7.4), 1% Triton X-100), supplemented with protease inhibitor cocktail (Roche Diagnostics) and DTT, Na_3_VO_4_, PMSF (each 1 mM), and NaF (20 mM). Thereafter, SDS-PAGE and immunoblotting was performed (50 µg of total protein per lane) using a goat polyclonal antibody (R&D Systems, #AF1034). In accord with previous data 26, murine CtsC protein produced specific staining at 25 kD.

### Statistical analysis

Data were first evaluated using the D´Agostino and Pearson test for parametric distribution. In case of 'n < 8' in at least one of the respective experimental groups, the Kolmogorov-Smirnov test was used for the whole experimental setup. Throughout the manuscript, 'n-numbers' indicate individual mice analyzed in a particular experimental setup. To compare two groups, raw data were evaluated by unpaired two-tailed Student's *t*-test or by Mann-Whitney-U-test, respectively. To compare three or more groups, raw data were analyzed by one-way analysis of variance (ANOVA) with post hoc Bonferroni correction or by Kruskal-Wallis test with post hoc Dunn's test. Statistical analyses are outlined in the Figure legends. Data are shown as means ± SEM (normally distributed data), as raw data points, or as box-plots (with top and bottom margins referring to the 75th and 25th percentile, with whiskers depicting the maximum and minimum values, and a horizontal line indicating the median; not normally distributed data) and presented as [% of total liver section], or as raw data (specifically, [target gene expression normalized to Gapdh], Units/L, pg/mL, ng/mL, ng/mg, μg/mg, μmol/g and [-log10 (FDR q-value)]). Differences are regarded statistically significant if p-values are less than 0.05 (GraphPad Prism 9, CA, USA). Linear regression analysis for ALT (Units/L) versus serum NSPs and associated coefficients of determination (R^2^) were calculated by simple linear regression analysis (GraphPad Prism 9).

## Results

### Analysis of splenic mRNA accumulation relating to acute inflammation during APAP-induced ALI

The spleen displays a context-specific regulatory potential that may affect course and consequences of systemic inflammation 41. In order to characterize splenic gene expression during the evolving resolution phase of APAP intoxication 16, mice were exposed to APAP for 30 h. Thereafter, serum ALT (Figure 1A), hepatic Cxcl2 gene expression (Figure 1B), the hepatic Cxcl2/ALT relationship (Figure 1C) as well as splenic Cxcl2 (Figure 1D) and TNFα (Figure 1E) gene expression were determined to verify liver injury and local/systemic inflammation. Absence of splenic Cxcl2 (Figure 1D) and TNFα (Figure 1E) gene induction indicates lack of manifest systemic inflammation at this interface time point (30 h) connecting hepatic injury and initial resolution/regeneration 16. Upregulation of hepatic Cxcl2 expression correlated (R^2^ = 0.6969, p < 0.0001) with liver damage detected by ALT (Figure 1B-C) which reflects ongoing local necroinflammation after APAP overdosing. Notably, increased hepatic, but not splenic, CtsC mRNA expression was likewise observed in these same samples suggesting that brisk inflammation may enhance CtsC in the liver (Figure S1A-B). Immunoblot analysis at that same time point after APAP administration showed a trend towards increased hepatic CtsC protein during intoxication (Figure S1C). For a broader picture, splenic tissue RNA specimens derived from mice shown in Figure 1A-E were used for 3′mRNA sequencing (MACE). For that purpose, RNA specimens (vehicle (0.9% NaCl)-treated, n = 5; APAP treated, n = 11) were pooled (per group) at equal shares. For data analysis, a threshold of '> 3-fold gene induction' was deployed creating a set of 302 differentially expressed genes. Of those were 46 genes that could be categorized based on their 'MACE gene fold-induction' in selected 'GO biological processes' (https://www.uniprot.org) that relate to acute inflammation (Figure S2A). Results obtained are consistent with mild systemic inflammation after APAP overdosing. Interestingly, splenic mRNA accumulation for NSPs popped up (Figure S2A) which concurs with myeloid cell mobilization during APAP-induced ALI 7 and may reflect splenic extramedullary hematopoiesis upregulated by inflammation 41. Splenic NSP gene induction during APAP intoxication was confirmed by additional experimentation evaluating the 24 h time point (Figure S2B-D). Moreover, significantly increased serum levels of CtsG (Figure 1F), NE (Figure 1G), and Prtn3 (Figure 1H) were detectable after APAP administration (analyzed at time point 30 h) - which likewise agrees with neutrophil and monocyte activation during intoxication 7. Interestingly, individual serum levels of CtsG (R^2^ = 0.8763, p < 0.0001) (Figure 1I), NE (R^2^ = 0.7089, p < 0.0001) (Figure 1J), and Prtn3 (R^2^ = 0.7354, p < 0.0001) (Figure 1K) strongly correlated with their linked serum ALT concentrations suggesting that neutrophil activation may relate to the severity of hepatic necrosis.

### CtsC deficient mice display ameliorated APAP-induced ALI

**Figure 2:**
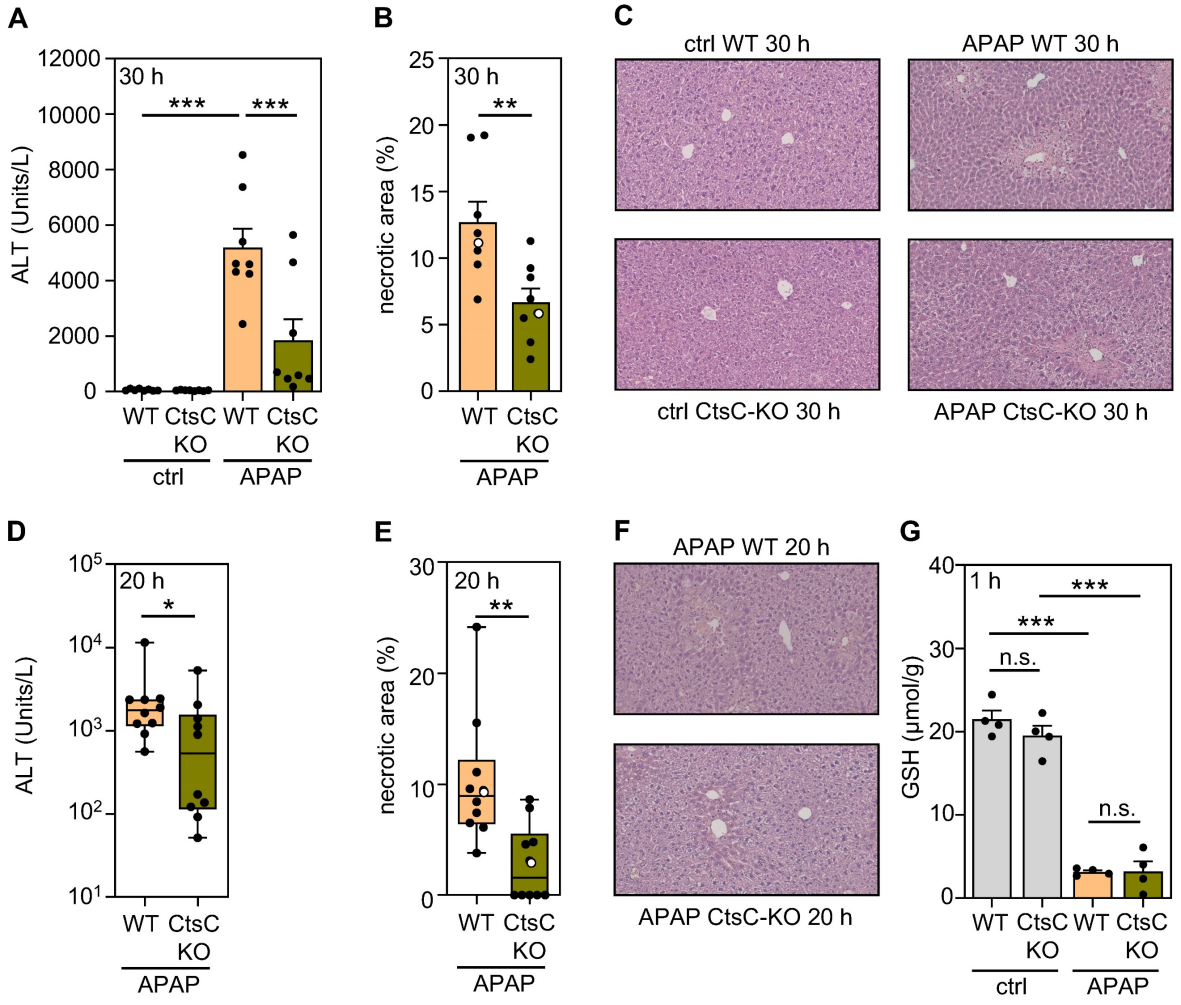
** CtsC-deficient mice display ameliorated APAP-induced ALI.** Male WT and CtsC-deficient (CtsC-KO) C57BL/6J mice received either 0.9% NaCl (ctrl) or APAP (300 mg/kg) (**A-C, G**) or APAP (300 mg/kg) (**D-F**). After 30 h (protocol I) (**A-C**), 20 h (protocol II) (**D-F**), or 1 h (**G**) hepatic tissue and serum was analyzed. **A, D** Serum ALT levels were measured after 30 h (**A**, n = 8 for each group; ***p < 0.001) or 20 h (**D**, n = 10 for each group; *p < 0.05). **B, E** Quantitative analysis of necrotic areas in H&E-stained liver sections obtained from APAP-treated mice at 30 h (**B**,* n* = 8 for each group; ** p < 0.01) or 20 h (**E**, *n* = 10 for each group; ** p < 0.01). **C, F** Representative images of H&E-stained sections at 30 h (**C**, ctrl and APAP-treated groups) or 20 h (**F**, APAP-treated groups). Selected specimens from APAP-treated groups shown as representative are highlighted as white circles in the corresponding subfigures **(B, E)**. **G** Levels of GSH were determined in liver homogenates (*n* = 4 for each group; ***p < 0.001). Statistical analysis: **A, G,** raw data were analyzed by one-way ANOVA with post hoc Bonferroni correction and are shown as means ± SEM; **B,** raw data were analyzed by unpaired Student´s *t*-test and are shown as means ± SEM; **D-E,** raw data were analyzed by Mann-Whitney-U-test and are shown as boxplots. n.s., not statistically significant.

In order to characterize functional consequences of CtsC deficiency, CtsC-KO mice were exposed to APAP at 300 mg/kg for 30 h (protocol I). Figure 2A-C demonstrates significant reduction of APAP-induced ALI in CtsC-KO mice which became apparent on the level of serum ALT (A) and histological parenchymal necrosis (B-C). Amelioration of APAP intoxication by CtsC deficiency was confirmed at the earlier 20 h time point (protocol II), again on the level of ALT (Figure 2D) and parenchymal necrosis (Figure 2E-F). Cyp1A2/Cyp2E1-dependent metabolization of APAP to NAPQI is key to intoxication and reflected by a sharp and rapid decline in hepatic levels of GSH 5, 6, 42. Analysis of hepatic GSH concentrations at 1 h after APAP administration demonstrated that the characteristic drop of this surrogate of APAP metabolism was unaltered in CtsC-KO mice (Figure 2G). Taken together, data presented reveal protection from APAP intoxication by chronic CtsC deficiency which is mechanistically unrelated to APAP metabolism.

### Therapeutic and prophylactic AZD7986 treatment relieves APAP-induced ALI

**Figure 3:**
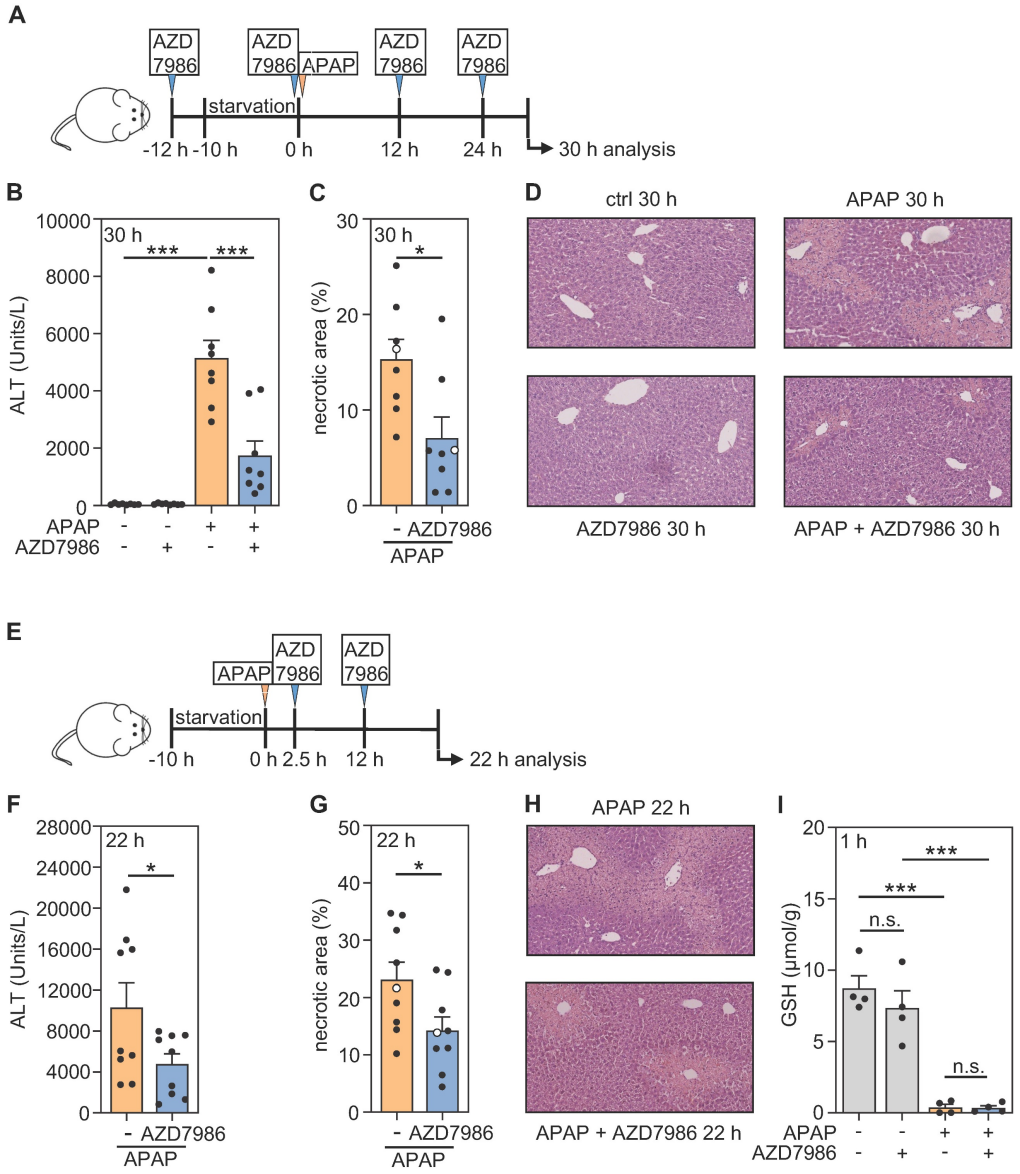
** Prophylactic and therapeutic administration of AZD7986 ameliorates APAP-induced ALI. A-D** Male WT C57BL/6J mice received, in a prophylactic treatment protocol with four AZD7986 administrations (**A**), either 0.9% NaCl (ctrl), or 0.9% NaCl + AZD7986 (5 mg/kg), or APAP (300 mg/kg), or APAP (300 mg/kg) + AZD7986 (5 mg/kg). Groups not treated with AZD7986 received four administrations of vehicle-AZD7986. After 30 h (protocol III), liver injury was determined by analysis of serum ALT (**B,** n = 8 for each group; ***p < 0.001) and by quantitative analysis of necrotic areas in H&E-stained liver sections (**C**, n = 8 for each group; *p < 0.05). **D** Representative images of H&E-stained sections. Selected specimens of APAP-treated groups shown as representative are highlighted as white circles in the corresponding subfigure **C**. **E-H** Male WT C57BL/6J mice received, in a therapeutic treatment protocol with two AZD7986 administrations (**E**), either APAP (300 mg/kg) + vehicle-AZD7986, or APAP (300 mg/kg) + AZD7986 (5 mg/kg). After 22 h (protocol IV), liver injury was determined by analysis of serum ALT (**F,** n = 9 for each group; *p < 0.05) and by quantitative analysis of necrotic areas in H&E-stained liver sections (**G**, n = 9 for each group; *p < 0.05). **H** Representative images of H&E-stained sections. Selected specimens of APAP-treated groups shown as representative are highlighted as white circles in the corresponding subfigure **G**. **I** Male WT C57BL/6J mice received, in a prophylactic treatment model with two AZD7986 administrations (**A**, with termination of experiment 1 h after APAP), either 0.9% NaCl (ctrl), or 0.9% NaCl + AZD7986 (5 mg/kg), or APAP (300 mg/kg), or APAP (300 mg/kg) + AZD7986 (5 mg/kg). Groups not treated with AZD7986 received two vehicle-AZD7986 administrations. AZD7986 or vehicle-AZD7986 were applied 12 h before and together with APAP. 1 h after APAP administration levels of GSH were determined in liver homogenates (*n* = 4 for each group; ***p < 0.001). Statistical analysis: **B, I**, raw data were analyzed by one-way ANOVA with post hoc Bonferroni correction and are shown as means ± SEM; **C, F, G,** raw data were analyzed by unpaired Student´s *t*-test and are shown as means ± SEM; n.s., not statistically significant.

AZD7986, also known as brensocatib, is a highly potent, reversible, and selective inhibitor of CtsC displaying a broad species-independent capacity for CtsC inhibition. Of note, the compound has been used successfully *in vivo* in healthy rodents (including C57BL/6 mice) and rodent disease models (different from ALI) as well as in human phase I and II clinical trials 35, 43-48. In order to investigate whether short-term application of this compound can affect the course of APAP-induced ALI, AZD7986 was first investigated in a prophylactic model comprising four administrations before and during ongoing intoxication (Figure 3A; protocol III). Interestingly, significant reduction of APAP-induced ALI was achieved by this protocol which became apparent on the level of serum ALT (Figure 3B) and histological parenchymal necrosis (Figure 3C-D). In order to further assess the translational potential of AZD7986 for treating ALI, the compound was additionally applied in a therapeutic protocol (protocol IV) 2.5 h and 12 h *post*-APAP (Figure 3E). Again, AZD7986 significantly ameliorated APAP intoxication as detected by serum ALT (Figure 3F) and histological necrosis (Figure 3G-H), respectively. Figure 3I furthermore demonstrates that AZD7986 did not influence APAP metabolization as analyzed 1 h after APAP administration (by determining of hepatic GSH levels) in the prophylactic treatment protocol. Data presented put forward the unexpected observation that short-term treatment with AZD7986, in a prophylactic or therapeutic protocol, mitigates APAP-induced ALI.

### Analysis of hepatic gene expression during APAP intoxication as detected in AZD7986-treated or CtsC-deficient mice

**Figure 4:**
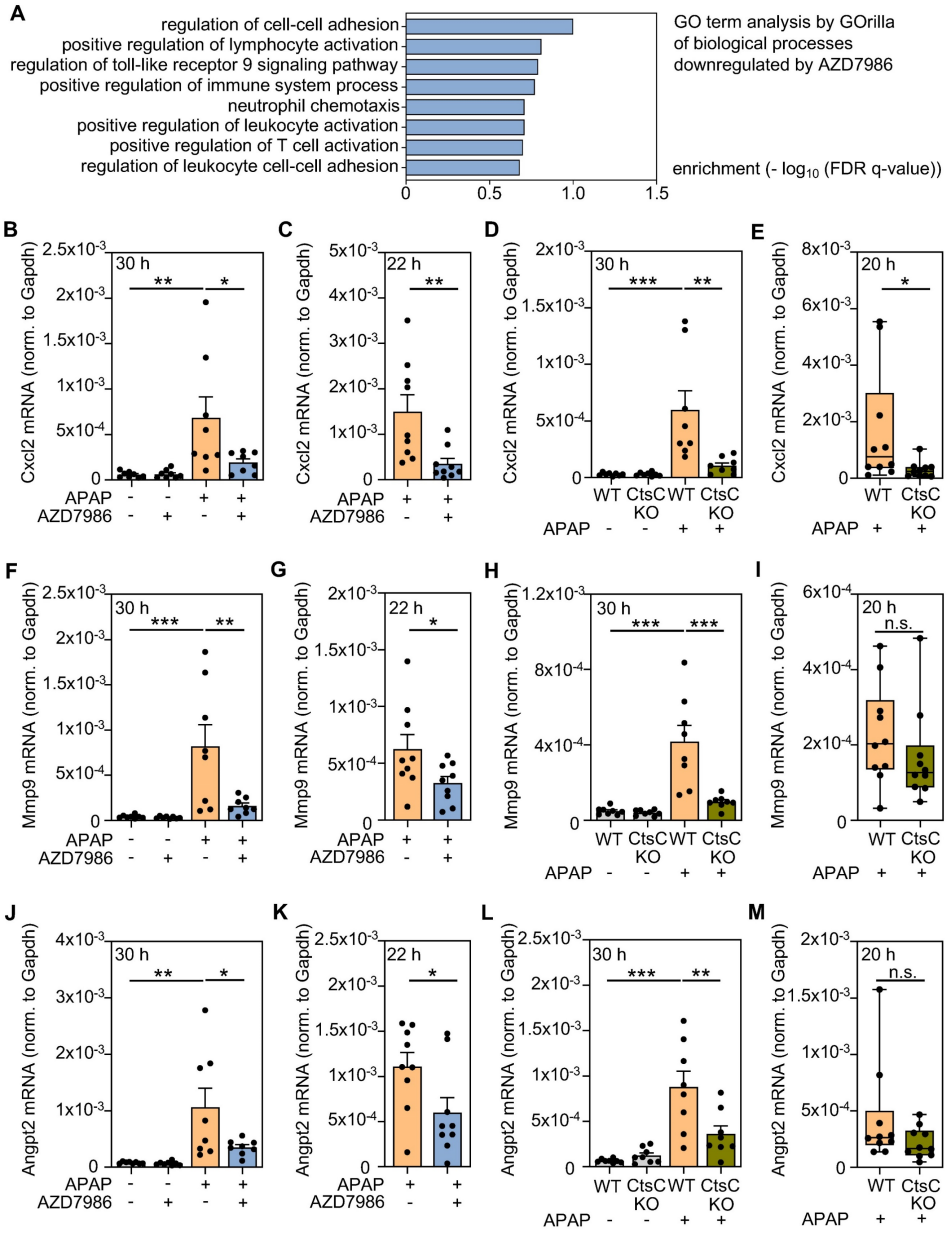
** Analysis of hepatic gene expression during APAP-induced ALI as detected under the influence of AZD7986 or in CtsC-deficient mice. A-B, F, J** Male WT C57BL/6J mice received, in the prophylactic treatment protocol III (n = 8 for each group) (see Figure 3A), either 0.9% NaCl (ctrl), or 0.9% NaCl + AZD7986 (5 mg/kg), or APAP (300 mg/kg), or APAP (300 mg/kg) + AZD7986 (5 mg/kg). Groups not treated with AZD7986 received four administrations of vehicle-AZD7986. **A** After 30 h (protocol III), hepatic RNA was pooled (per experimental condition) in equal shares and evaluated for gene expression by MACE. GO term analysis was performed on 2135 genes significantly downregulated by AZD7986 in APAP-treated mice (by at least 50%) using GOrilla. Shown are biological processes significantly downregulated (p < 0.001) by AZD7986 that relate to necroinflammation/immunoregulation. **B, F, J** After 30 h, hepatic mRNA expression of Cxcl2 (**B**), Mmp9 (**F**), and Angpt2 (**J**) was quantified using realtime PCR. Target mRNA normalized to Gapdh is shown as absolute values (*p < 0.05, **p < 0.01, ***p < 0.001). **C, G, K** Male WT C57BL/6J mice received, in the therapeutic treatment protocol (see Figure 3E), either APAP (300 mg/kg) + vehicle-AZD7986, or APAP (300 mg/kg) + AZD7986 (5 mg/kg). After 22 h (protocol IV), hepatic mRNA expression of Cxcl2 (**C**), Mmp9 (**G**), and Angpt2 (**K**) was quantified using realtime PCR. Target mRNA normalized to Gapdh is shown as absolute values (n = 9 for each group; *p < 0.05, **p < 0.01). **D-E, H-I, L-M** Male WT and CtsC-KO C57BL/6J mice received either 0.9% NaCl (ctrl) or APAP (300 mg/kg) with n = 8 for each group (**D, H, L**) or APAP (300 mg/kg) with n = 10 for each group (**E, I, M**) (see Figure 2). After 30 h (**D, H, L**, protocol I) or 20 h (**E, I, M**, protocol II), hepatic mRNA expression of Cxcl2 (**D-E**), Mmp9 (**H-I**), and Angpt2 (**L-M**) was quantified using realtime PCR. Target mRNA normalized to Gapdh is shown as absolute values (*p < 0.05, **p < 0.01, ***p < 0.001). Statistical analysis: **B, D, F, H, J, L,** raw data were analyzed by one-way ANOVA with post hoc Bonferroni correction and are shown as means ± SEM; **C, G, K,** raw data were analyzed by unpaired Student´s *t*-test and are shown as means ± SEM; **E, I, M,** raw data were analyzed by Mann-Whitney-U-test and are shown as boxplots; n.s., not statistically significant.

In order to gain a broader picture of AZD7986 effects on hepatic gene expression, RNA specimens from all APAP-treated mice used in the prophylactic model (Figure 3A-D) were pooled (per group) at equal shares. Thereafter, pooled samples were analyzed by MACE with focus on genes inhibited under the influence of AZD7986. After applying a threshold of 'at least 50% downregulation by AZD7986', a set of 2135 genes evolved that was further evaluated based on GOrilla analysis 40 or based on documented involvement in ALI and/or liver failure. Figure 4A displays significantly enriched (p < 0.001) selected GO terms (downregulated by AZD7986) that relate to the process of necroinflammation - a critical parameter of APAP intoxication 8-13. A complete list of those enriched GO terms is shown in Table S1. This approach revealed several inflammatory parameters including a variety of candidate genes previously reported to be pathogenic in diverse forms of ALI. Those were further analyzed in the four treatment protocols applied in Figure 2 and Figure 3 (CtsC-KO mice, time point 30 h (protocol I) and 20 h (protocol II); prophylactic (protocol III; analysis after 30 h) and therapeutic (protocol IV; analysis after 22 h) AZD7986-treatment). For that purpose, hepatic gene expression was evaluated in RNA samples of individual mice by realtime PCR. Thereby, we identified three genes previously associated with ALI/acute liver failure that were significantly downregulated in at least three out of the four protocols tested and showed a discernable tendency towards inhibition in a fourth protocol. Those were *cxcl2* 17, 22 (Figure 4B**-**E), *mmp9* 49 (Figure 4F-I), and *angpt2* 50 (Figure 4J**-**M).

**Figure 5:**
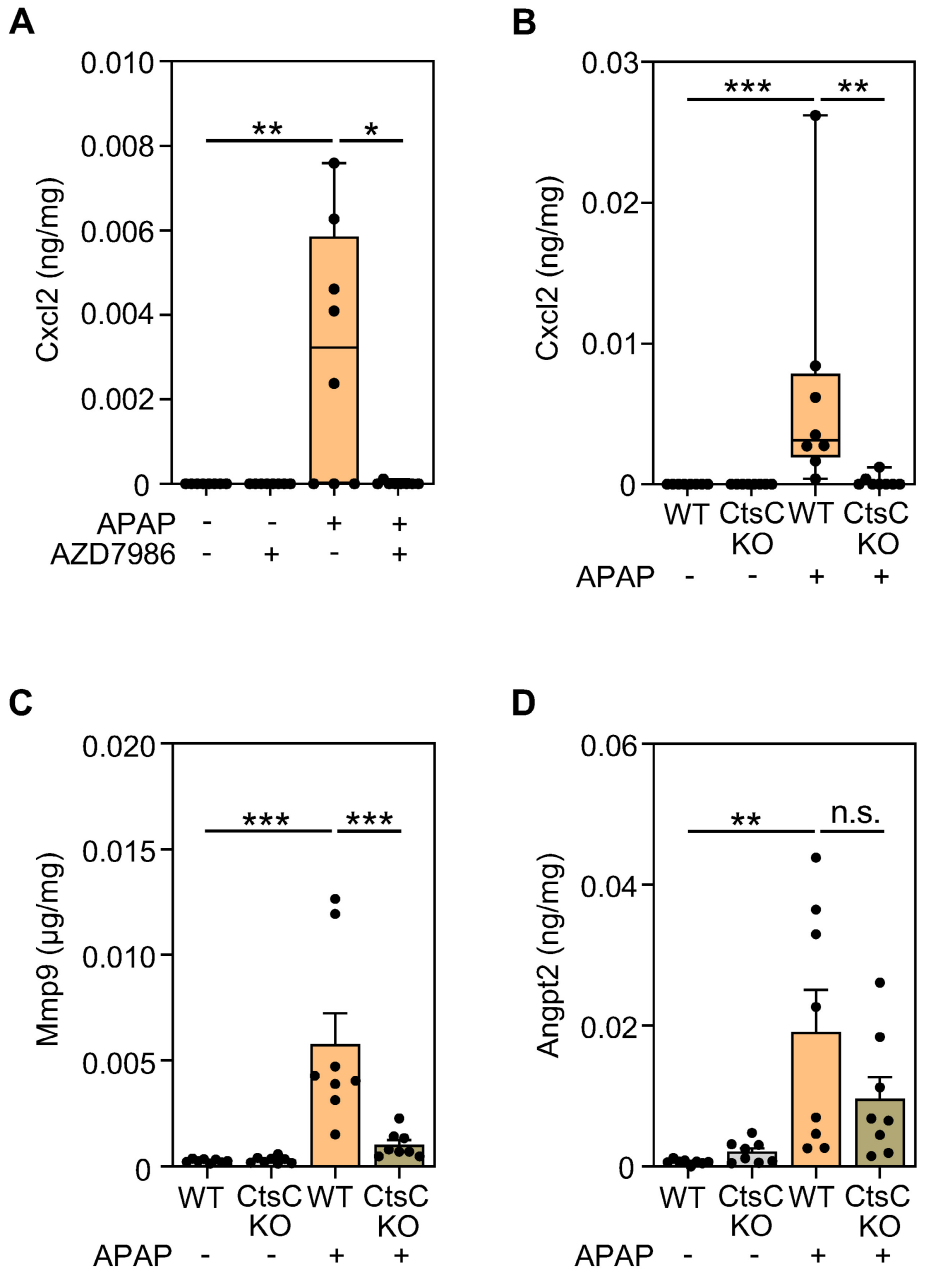
** Modulation of Cxcl2, Mmp9, and Angpt2 protein expression by targeting CtsC. A** Male WT C57BL/6J mice received, in a prophylactic treatment protocol with four AZD7986 administrations (see Figure 3A), either 0.9% NaCl (ctrl), or 0.9% NaCl + AZD7986 (5 mg/kg), or APAP (300 mg/kg), or APAP (300 mg/kg) + AZD7986 (5 mg/kg). Groups not treated with AZD7986 received four administrations of vehicle-AZD7986. After 30 h (protocol III), hepatic protein levels of Cxcl2 were analyzed by ELISA (n = 8 for each group; *p < 0.05, **p < 0.01). **B-D** Male WT and CtsC-KO C57BL/6J mice received either 0.9% NaCl (ctrl) or APAP (300 mg/kg) (see Figure 2A-C). After 30 h (protocol I), hepatic levels of Cxcl2 (**B**), Mmp9 (**C**), and Angpt2 (**D**) protein were analyzed by ELISA (n = 8 for each group; **p < 0.01, ***p < 0.001). Statistical analysis: **A-B**, raw data were analyzed by Kruskal-Wallis test with post hoc Dunn's test and are shown as boxplots; **C-D**, raw data were analyzed by one-way ANOVA with post hoc Bonferroni correction and are shown as means ± SEM. n.s., not statistically significant.

Hepatic mRNA data translated to the protein levels as shown for Cxcl2 which was analyzed using protocol III (Figure 5A) and I (Figure 5B) and for Mmp9 (Figure 5C) which was analyzed using protocol I. Angpt2 protein (Figure 5D), likewise analyzed in CtsC-KO mice using protocol I, showed a tendency towards downregulation that, however, did not reach statistical significance in the group of mice analyzed.

**Figure 6:**
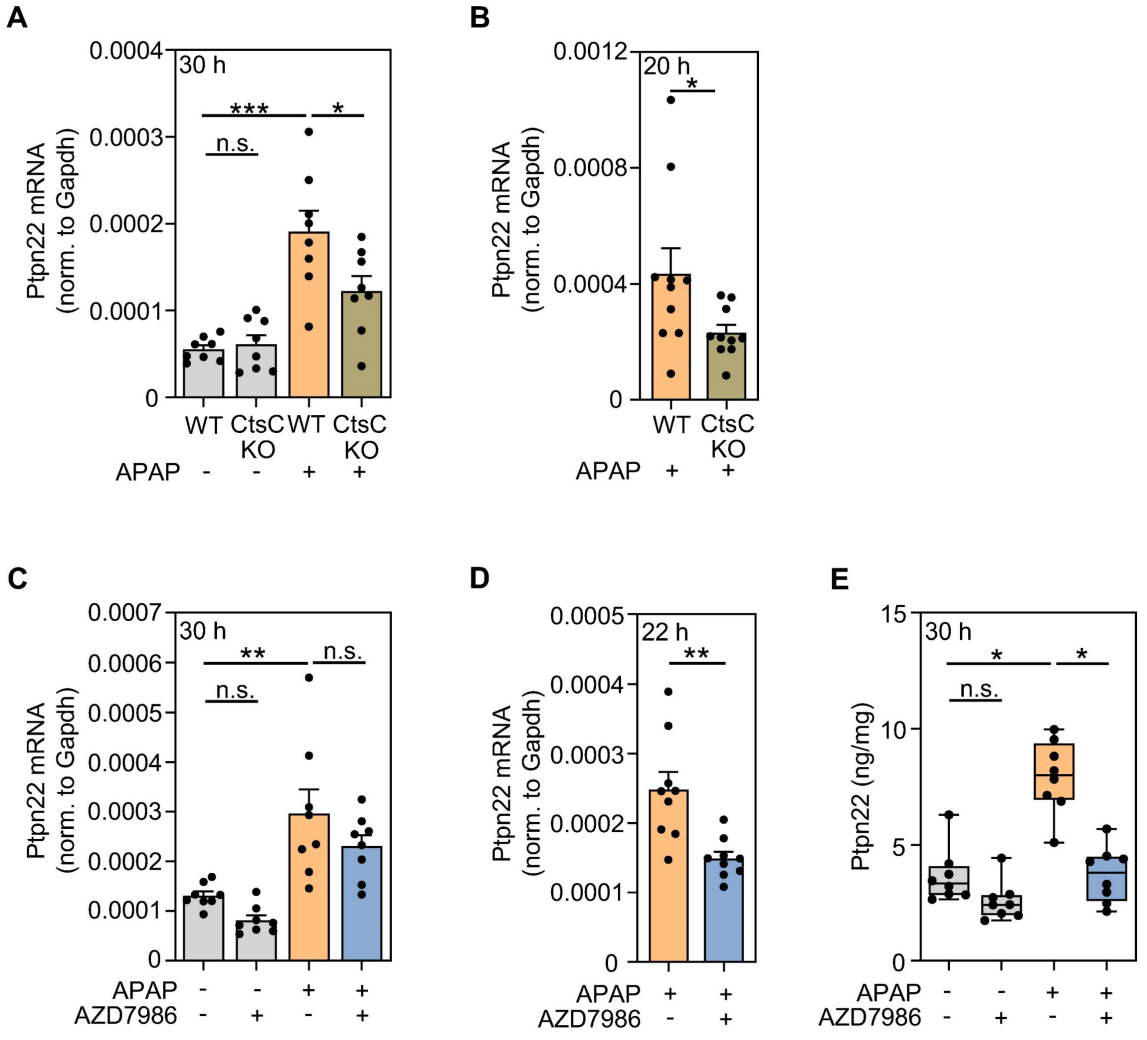
** Modulation of Ptpn22 expression by targeting CtsC. A-B** Male WT and CtsC-KO C57BL/6J mice received either 0.9% NaCl (ctrl) or APAP (300 mg/kg) (**A**) or APAP (300 mg/kg) (**B**) (see Figure 2A-F). After 30 h (protocol I) with n = 8 for each group (**A**) or 20 h with n = 10 for each group (protocol II) (**B**), hepatic mRNA expression of Ptpn22 was quantified using realtime PCR. Target mRNA normalized to Gapdh is shown as absolute values (*p < 0.05, ***p < 0.001). **C, E** Male wildtype C57BL/6J mice received, in a prophylactic treatment protocol with four AZD7986 administrations (see Figure 3A), either 0.9% NaCl (ctrl), or 0.9% NaCl + AZD7986 (5 mg/kg), or APAP (300 mg/kg), or APAP (300 mg/kg) + AZD7986 (5 mg/kg). Groups not treated with AZD7986 received four administrations of vehicle-AZD7986. **C** After 30 h (protocol III), hepatic mRNA expression of Ptpn22 was quantified using realtime PCR. Target mRNA normalized to Gapdh is shown as absolute values (n = 8 for each group; **p < 0.01). **D** Male WT C57BL/6J mice received, in the therapeutic treatment protocol (see Figure 3E), either APAP (300 mg/kg) + vehicle-AZD7986, or APAP (300 mg/kg) + AZD7986 (5 mg/kg). After 22 h (protocol IV), hepatic mRNA expression of Ptpn22 was quantified using realtime PCR. Target mRNA normalized to Gapdh is shown as absolute values (n = 9 for each group; **p < 0.01). **E** After 30 h (protocol III), hepatic protein levels of Ptpn22 were analyzed by ELISA (n = 8 for each group; *p < 0.05). Statistical analysis: **A, C**, raw data were analyzed by one-way ANOVA with post hoc Bonferroni correction and are shown as means ± SEM; **B, D**, raw data were analyzed by unpaired Student´s *t*-test and are shown as means ± SEM; **E**, raw data were analyzed by Kruskal-Wallis test with post hoc Dunn's test and are shown as boxplots; n.s., not statistically significant.

GOrilla analysis (Figure 4A) of genes downregulated by AZD7986 suggested significant regulation of the GO term 'regulation of toll-like receptor 9 signaling' and of the associated gene *ptpn22*. Ptpn22 is regarded a positive regulator of toll-like receptor-3, -4, -9-induced type I IFN production 51, 52. Interestingly, type I IFN receptor signaling aggravates APAP-induced ALI, particularly in the early phase of injury 53-55. Subsequent evaluation of RNA specimens derived from individual mice confirmed a significant reduction of *ptpn22* expression in protocol I (Figure 6A), II (Figure 6B), and IV (Figure 6D); only a tendency towards Ptpn22 modulation was observed for protocol III (Figure 6C). Of note, analysis of hepatic Ptpn22 protein expression by ELISA revealed significant inhibition of this phosphatase by AZD7986 at 30 h after APAP administration (protocol III) in these same mice.

## Discussion

CtsC activity is considered key to neutrophil differentiation thus directing the course of syndromes associated with acute inflammation, among others APAP-induced ALI. As already alluded to, the role of neutrophilic inflammation in APAP intoxication is complex in terms of function but can drive pathology in cases where the insult exceeds a threshold of hepatic tissue damage. Under those conditions of prevailing pathological inflammation, inhibition of neutrophil-activating Cxcl2 by neutralizing antibodies 22 or by Cxcr2 antagonism 17 can be protective. Aforementioned threshold levels of hepatic damage likely depend on complex parameters that may include intestinal permeability and the gut microbiota repertoire 56. Of note, pathological (myeloid) inflammation in APAP-induced ALI also extends to early infiltrating monocytes as evidenced by protection after targeting the Ccl2/Ccr2-axis 57.

Herein, we set out to characterize APAP-induced ALI in CtsC-KO mice and their WT counterparts. Reduced toxicity as observed in CtsC-KO mice was independent on APAP metabolization and detectable on the level of histological necrosis and serum ALT. Data fully agree with previously reported amelioration of APAP-induced ALI in mice deficient for *elane*/NE 18 and imply that downregulation of NSPs by chronic lack of CtsC activity is at the basis of hepatic protection seen in CtsC-KO mice.

Due to the fact that CtsC acts on early hematopoietic precursors, downregulation of NSPs by pharmacological means requires extended periods of CtsC inhibition for several days 35, 43. The translational potential of short-term CtsC inhibition in APAP intoxication was nevertheless evaluated by applying a prophylactic and a therapeutic AZD7986 treatment protocol. Surprisingly, short-term administration of AZD7986 reduced liver damage in response to APAP in both protocols. This observation suggests an additional more rapid mode of action responsible for hepatic protection by CtsC inhibition - beyond inhibition of granule-associated serine proteases. On the other hand, although AZD7986 is considered to be a highly potent and selective CtsC inhibitor 25, 48, it is impossible to fully exclude that hitherto unrecognized tissue-protective off-target effects of the compound are at work as contributing factor. Of note, amelioration of APAP-induced ALI by therapeutically administered AZD7986 was observed when given 2.5 h after APAP. Therefore, we can exclude that the compound acts on APAP metabolization because the given dosage of 300 mg/kg is already fully metabolized at this time point 42, 58.

Hepatic 3'mRNA sequencing combined with GOrilla analysis revealed that AZD7986 inhibited 'GO biological processes' that trigger acute inflammation in response to APAP. Further analysis of expression data disclosed a set of genes showing broad downregulation in CtsC-KO and AZD7986-treated-WT mice (protocols I-IV) along with documented pathogenic functions in ALI or liver failure, namely *cxcl2*, *mmp9*, and *angpt2.* Whereas Cxcl2 is key to initiation of aforementioned pathogenic inflammation 17, 22, Mmp9 and Angpt2 have critical functions in more distal parts of the inflammatory cascade. In fact, Mmp9 has been linked to pathogenesis in several rodent ALI models including APAP intoxication 49, lipopolysaccharide/β-galactosamine-induced ALI 59, and hepatic ischemia/reperfusion injury 60. In accord with data presented herein, overexpression of CtsC in human hepatoma cells has been associated with augmented Mmp9 61. Mechanistically, Mmp9 is supposed to contribute to ALI-associated sinusoidal demise and vascular pathology 49, 59. Mmp9 actually interferes with hepatic repair/resolution and regeneration by degrading vascular endothelial growth factor (VEGF) which is key to the recruitment of liver sinusoidal endothelial cell (LSEC) progenitor cells 62. Of note, VEGF signaling supports repair in murine APAP-induced ALI, particularly in later phases of intoxication 63, 64. Angpt2 has been described as surrogate of disease severity in clinical acute liver failure 65 and its relative upregulation (versus Angpt1) in murine acute-on-chronic-liver failure mediates hepatocyte dysfunction and disease via CCAAT/enhancer binding protein-β 50. Interestingly, Angpt2 can also support production of anti-proliferative transforming growth factor-β1 by LSEC which was demonstrated in murine partial hepatectomy 66. This observation should be relevant in the given context because transforming growth factor-β1 likewise restrains regeneration in APAP-induced ALI 67. Instructed by GOrilla analysis, we also investigated regulation of hepatic Ptpn22 expression. Herein, we introduce this primarily leukocytic phosphatase as inducible during ALI. In addition to amplifying innate production of type I IFN 51, 52, Ptpn22 has the capability to enhance IFN signal transduction which was demonstrated by analysis of signal transducer and activator of transcription-1 in IFNα-stimulated (Ptpn22-deficient) CD8^+^ T cells 68. Therefore, Ptpn22 can potentiate type I IFN biological activity on several levels which should promote liver injury after APAP overdosing 53-55. Taken together, genetic CtsC deficiency or treatment with AZD7986 consistently interfered with expression of Cxcl2, Mmp9, Angpt2, and Ptpn22 which are regarded pathogenic by action on the hepatic injury (up to 24 h after APAP 42) and/or the repair/resolution (after 24 h 42) phase of ALI.

As already alluded to, CtsC inhibition may promote hepatic repair and disease resolution e.g. by suppression of Mmp9. Of note, a recent study by Feng et al. adds complexity at this point. Data presented suggest that cathepsin B (CtsB) supports resolution in the late phase (96 h) of concanavalin A-induced liver injury 69. Because CtsC has been shown to increase activation of rodent CtsB, at least *in vitro* 70, a reduction of CtsC-dependent CtsB may modulate protective properties of AZD7986.

CtsC is constitutively expressed in various organs including spleen, lung, kidney, and liver 25 with active enzyme being released by activated myeloid cells such as neutrophils but also by some non-leukocytic cell types 25, 71. However, substrates and functions of CtsC beyond activation of granule-associated serine proteases in hematopoietic precursors are barely characterized. Recently, it was shown that renal podocytes release active CtsC which could be further augmented by exposure to high-glucose. Since CtsC downregulation in podocytes decreased their albumin permeability, podocyte-derived CtsC has been suggested to contribute to kidney injury in diabetic nephropathy 71. A potentially most interesting target of CtsC in APAP-induced ALI is the urokinase-type plasminogen activator. Intriguingly, CtsC has been shown to be capable of re-activating thrombin-cleaved urokinase-type plasminogen activator 72, 73. Since plasmin is pathogenic in APAP-induced ALI 74, 75, it is tempting to speculate that CtsC may act via augmented plasmin activation during early intoxication.

## Conclusions

Taken together, this work suggests CtsC as therapeutic target for the treatment of paradigmatic APAP-induced ALI. Since AZD7986 (brensocatib) is currently being evaluated in a phase III clinical trial for the treatment of non-cystic-fibrosis bronchiectasis, data presented are relevant in terms of translation and shed light on a novel therapeutic approach addressing drug-induced ALI in general.

## Supplementary Material

Supplementary figures and table.

## Abbreviations

ALI: acute liver injury
Angpt2: Angiopoietin-2
APAP: Acetaminophen
ctrl: Control
Cts: Cathepsin
Cxcl2: C-X-C motif chemokine 2
ELISA: Enzyme-linked immunosorbent assay
Gapdh: Glyceraldehyde-3-phoshate dehydrogenase
Glutathione (reduced): GSH
IL: Interleukin
IFN: Interferon
KO: knockout
MACE: Massive analysis of cDNA ends
MMP9: Matrix metalloproteinase-9
NAPQI: N-acetyl-para-benzoquinone imine
NE: Neutrophil elastase
NSP: Neutrophil serin protease
PCR: Polymerase chain reaction
Prtn3: Proteinase-3
Ptpn22: Tyrosine-protein phosphatase non-receptor type 22
Tnfα: Tumor necrosis factor-α
WT: wildtype
