# Supplementary material for: Targeting cathepsin C ameliorates murine acetaminophen-induced liver injury
Source: Theranostics. 2024 May 13;14(8):3029–42. doi: 10.7150/thno.96092 (PMC11155399; doi:10.7150/thno.96092)
Supplement: Supplementary file 1 — Supplementary figures and table. [file thnov14p3029s1.pdf]

Figure S1

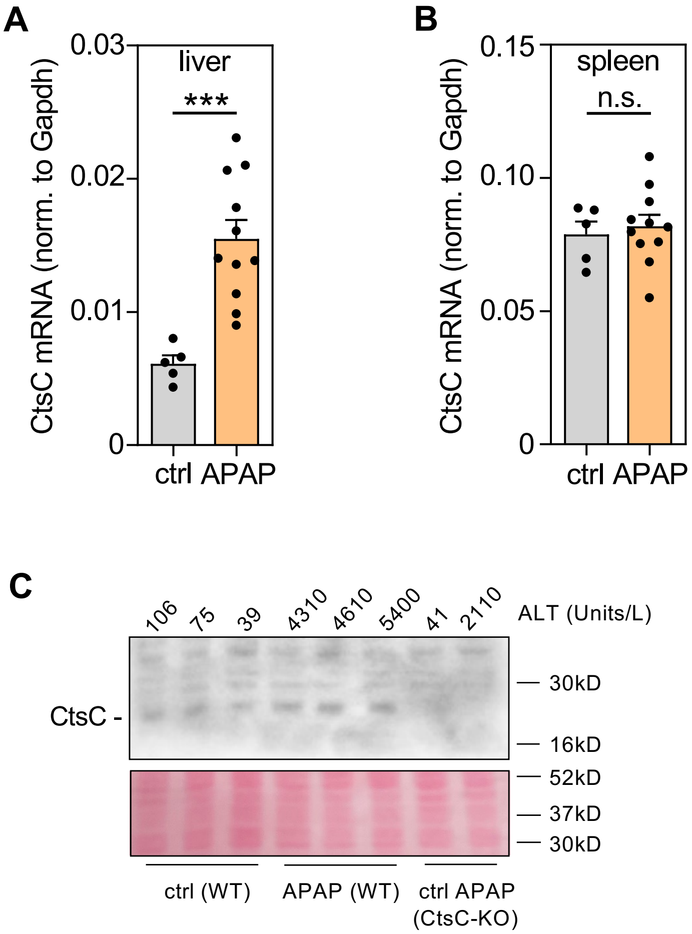
A
B
C
liver
spleen
***
n.s.
CtsC mRNA (norm. to Gapdh)
0.03
0.02
0.01
0
0.15
0.10
0.05
ctrl
APAP
106
75
39
4310
4610
5400
41
2110
ALT (Units/L)
CtsC -
30kD
16kD
52kD
37kD
30kD
ctrl (WT)
APAP (WT)
ctrl APAP
(CtsC-KO)

Legend Figure S1

CtsC gene expression in APAP-induced ALI. A–B Male C57BL/6J mice received either 0.9% NaCl (ctrl, n = 5) or APAP at 300 mg/kg (n = 11). After 30 h, splenic/hepatic tissues and sera were analyzed (see Fig. 1A–D). Splenic (A) and hepatic (B) mRNA expression of CtsC in these same mice was analyzed using realtime PCR. Target mRNA normalized to Gapdh is shown as absolute values (***$p < 0.001$). **C** Male WT and CtsC-deficient (CtsC-KO) C57BL/6J mice received either 0.9% NaCl (ctrl) or APAP (300 mg/kg). After 30 h (protocol I) hepatic tissue and serum was analyzed (see Figure 2A–C**)**. Mouse specimens were randomly selected for immunoblot analysis of CtsC. Linked ALT levels are additionally shown. In accord with previous data [26], murine CtsC protein produced a specific band at 25 kD which, as expected, disappeared in CtsC-KO mice. In addition, a Ponceau S stain of the blot is shown. Statistical analysis: raw data were analyzed by unpaired Student´s t-test and are shown as means ± SEM; n.s., not statistically significant.

Figure S2

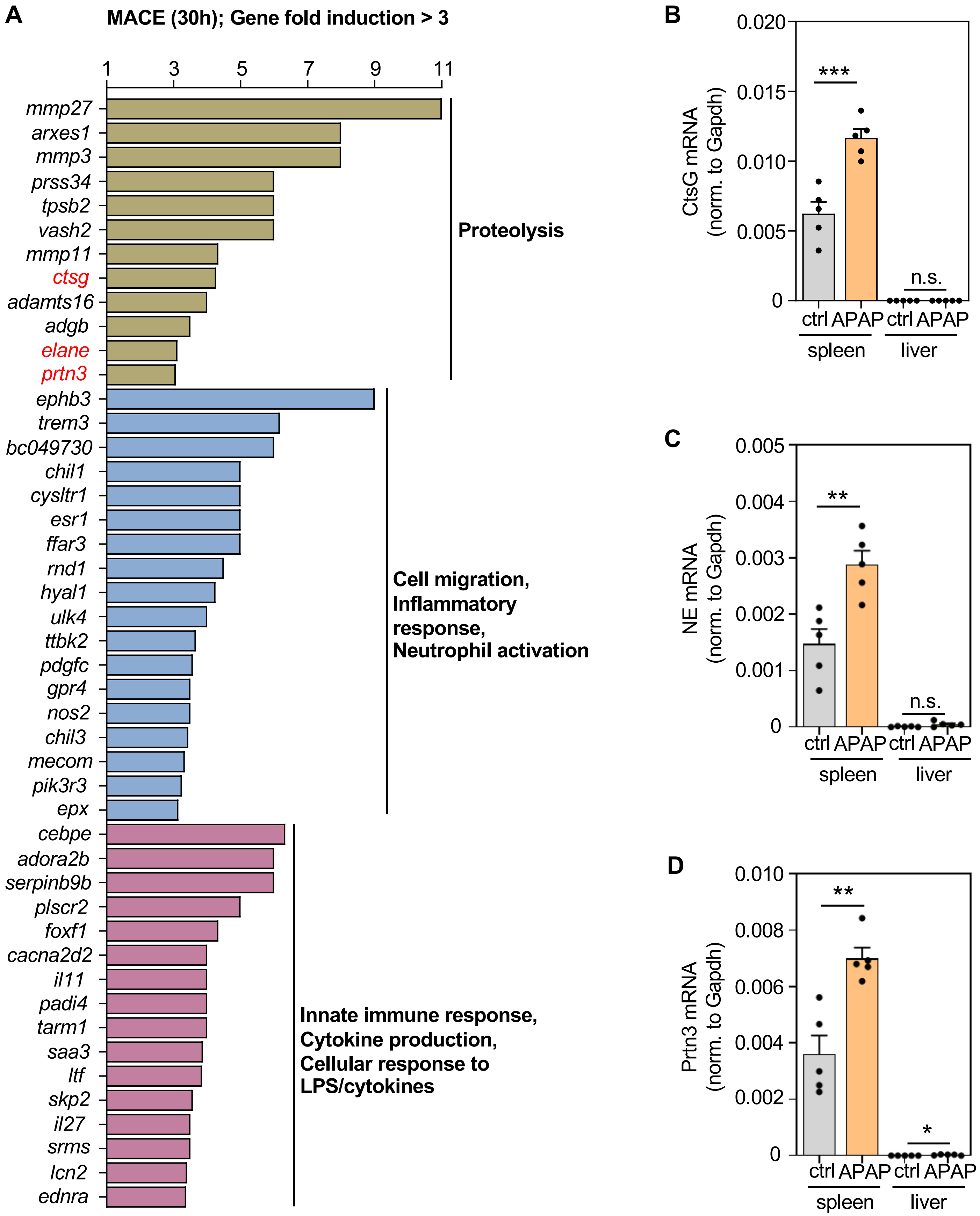

A
MACE (30h); Gene fold induction > 3
1
3
5
7
9
11
mmp27
arxes1
mmp3
prss34
tpsb2
vash2
mmp11
ctsg
adamts16
adgb
elane
prtn3
Proteolysis
ephb3
trem3
bc049730
chil1
cysltr1
esr1
ffar3
rnd1
hyal1
ulk4
ttbk2
pdgfc
gpr4
nos2
chil3
mecom
pik3r3
epx
Cell migration,
Inflammatory
response,
Neutrophil activation
cebpe
adora2b
serpinb9b
plscr2
foxf1
cacna2d2
il11
padi4
tarm1
saa3
ltf
skp2
il27
srms
lcn2
ednra
Innate immune response,
Cytokine production,
Cellular response to
LPS/cytokines
B
CtsG mRNA
(norm. to Gapdh)
0.020
0.015
0.010
0.005
0
***
n.s.
ctrl APAP ctrl APAP
spleen
liver
C
NE mRNA
(norm. to Gapdh)
0.005
0.004
0.003
0.002
0.001
0
**
n.s.
ctrl APAP ctrl APAP
spleen
liver
D
Prtn3 mRNA
(norm. to Gapdh)
0.010
0.008
0.006
0.004
0.002
0
**
*
ctrl APAP ctrl APAP
spleen
liver

Legend Figure S2

Analysis of inflammatory splenic gene expression after APAP administration. A Male WT C57BL/6J mice received 0.9% NaCl (ctrl, n = 5) or APAP at 300 mg/kg (n = 11). After 30h, experiments were terminated, and splenic/hepatic RNA was isolated. Splenic RNA was pooled (per experimental condition) in equal shares and global gene expression was evaluated by MACE as outlined in the methods section. Analysis disclosed gene induction of 302 genes (threshold: 3-fold gene induction by APAP treatment). Of those, 46 genes are depicted that could be grouped based on their 'MACE gene fold-induction' in selected 'GO biological processes' (https://www.uniprot.org) that relate to acute inflammation. **B–D** Male WT C57BL/6J mice received 0.9% NaCl (ctrl, n = 5) or APAP at 300 mg/kg (n = 5). These mice received two additional i.p. injections that were required for vehicle control in the context of a different study: one PBS injection 1h before APAP (or 0.9% NaCl as APAP vehicle) and one injection of 0.9% NaCl 2h after APAP (or 0.9% NaCl as APAP vehicle). 24h after APAP administration, splenic and hepatic mRNA expression of CtsG (**B**), NE (**C**), and Prtn3 (**D**) was verified using realtime PCR. Target mRNA normalized to Gapdh is shown as absolute values (*p < 0.05, **p < 0.01, ***p < 0.001). Statistical analysis: **B** (spleen) **CD**, raw data were analyzed by unpaired Student´s *t*-test (ctrl *versus* APAP for spleen or liver, respectively) and are shown as means ± SEM; **B** (liver), raw data were analyzed by Mann-Whitney-U-test and are shown as raw data points; n.s., not statistically significant.

Table S1

GO Term	Description	P-value	FDR q-value	- log $_{10}$ (FDR q-value)
GO:0036342	post-anal tail morphogenesis	3.43E-6	3.12E-2	1.505845406
GO:0022409	positive regulation of cell-cell adhesion	1.4E-5	6.35E-2	1.197226275
GO:1903039	positive regulation of leukocyte cell-cell adhesion	3.53E-5	8.01E-2	1.096367484
GO:0022407	regulation of cell-cell adhesion	3.31E-5	1.00E-01	1
GO:1900044	regulation of protein K63-linked ubiquitination	4.96E-4	1.5E-1	0.823908741
GO:0050867	positive regulation of cell activation	5.13E-4	1.5E-1	0.823908741
GO:0034165	positive regulation of toll-like receptor 9 signaling pathway	4.96E-4	1.55E-1	0.809668302
GO:1904938	planar cell polarity pathway involved in axon guidance	5.5E-4	1.56E-1	0.806875402
GO:0051251	positive regulation of lymphocyte activation	5.67E-4	1.56E-1	0.806875402
GO:0034163	regulation of toll-like receptor 9 signaling pathway	4.96E-4	1.61E-1	0.793174124
GO:1902914	regulation of protein polyubiquitination	4.96E-4	1.67E-1	0.777283529
GO:0002684	positive regulation of immune system process	6.32E-4	1.69E-1	0.772113295
GO:1902916	positive regulation of protein polyubiquitination	4.96E-4	1.73E-1	0.761953897
GO:1990266	neutrophil migration	6.8E-4	1.77E-1	0.752026734
GO:1902523	positive regulation of protein K63-linked ubiquitination	4.96E-4	1.8E-1	0.744727495
GO:0070425	negative regulation of nucleotide-binding oligomerization domain containing signaling pathway	4.96E-4	1.88E-1	0.725842151
GO:0045785	positive regulation of cell adhesion	2.77E-4	1.93E-1	0.714442691
GO:0030593	neutrophil chemotaxis	2.36E-4	1.94E-1	0.71219827
GO:0007411	axon guidance	3.86E-4	1.95E-1	0.709965389
GO:0070424	regulation of nucleotide-binding oligomerization domain containing signaling pathway	4.96E-4	1.96E-1	0.707743929
GO:0002696	positive regulation of leukocyte activation	3.25E-4	1.97E-1	0.705533774
GO:0050870	positive regulation of T cell activation	2.19E-4	1.99E-1	0.701146924
GO:0051445	regulation of meiotic cell cycle	2.64E-4	1.99E-1	0.701146924
GO:0034501	protein localization to kinetochore	3.58E-4	2.03E-1	0.692503962
GO:0070433	negative regulation of nucleotide-binding oligomerization domain containing 2 signaling pathway	4.96E-4	2.05E-1	0.688246139
GO:0097485	neuron projection guidance	3.86E-4	2.06E-1	0.68613278
GO:0033598	mammary gland epithelial cell proliferation	2.07E-4	2.09E-1	0.679853714
GO:1903037	regulation of leukocyte cell-cell adhesion	3.25E-4	2.11E-1	0.675717545
GO:0070432	regulation of nucleotide-binding oligomerization domain containing 2 signaling pathway	4.96E-4	2.15E-1	0.66756154
GO:0035644	phosphoanandamide dephosphorylation	4.96E-4	2.25E-1	0.647817482
GO:0060744	mammary gland branching involved in thelarche	2.07E-4	2.35E-1	0.628932138
GO:0050860	negative regulation of T cell receptor signaling pathway	4.96E-4	2.37E-1	0.625251654
GO:0060750	epithelial cell proliferation involved in mammary gland duct elongation	2.07E-4	2.68E-1	0.571865206
GO:0045621	positive regulation of lymphocyte differentiation	1.92E-4	2.91E-1	0.536107011
GO:0045582	positive regulation of T cell differentiation	1.92E-4	3.49E-1	0.457174573
